# Propionic Acid Outperforms Formic and Acetic Acid in MS Sensitivity for High-Flow Reversed-Phase LC-MS Bottom-Up Proteomics

**DOI:** 10.1021/acs.analchem.5c07595

**Published:** 2026-04-03

**Authors:** Mykyta R. Starovoit, Siddharth Jadeja, Rudolf Kupčík, Saša Vatić, Jan Rasl, Derya Demir, Petr Novák, Cameron Braswell, Benjamin C. Orsburn, Juraj Lenčo

**Affiliations:** † Department of Analytical Chemistry, Faculty of Pharmacy in Hradec Králové, 69727 Charles University, Heyrovského 1203/8, Hradec Králové 500 03, Czech Republic; ‡ Biomedical Research Centre, University Hospital Hradec Králové, Sokolská 581, Hradec Králové 500 05, Czech Republic; § Laboratory of Structural Biology and Cell Signaling, 86863 Institute of Microbiology, Czech Academy of Sciences, BioCeV, Vídeňská 1083, Prague 4 142 00, Czech Republic; ∥ Department of Biochemistry, Faculty of Science, Charles University, Hlavova 6, Prague 2 12843, Czech Republic; ⊥ Organ Pathobiology and Therapeutics Institute, 6614 University of Pittsburgh, Pittsburgh, Pennsylvania 15203, United States

## Abstract

Formic acid has long been the default acidic additive in reversed-phase LC-MS-based bottom-up proteomics, offering a practical balance between chromatographic performance and electrospray ionization (ESI) efficiency. Here, we evaluate propionic acid as an alternative mobile phase acidifier, a candidate that has been largely overlooked in efforts to improve ESI efficiency without compromising chromatography. By reducing both the ionic strength and surface tension of the mobile phase, propionic acid markedly enhanced ESI efficiency, yielding an average 39% increase in peptide identifications compared to formic acid and even a 12% increase relative to the recently revived acetic acid. These gains were consistent across interlaboratory data sets encompassing analytical- and microflow LC-MS configurations, diverse column chemistries, and varying sample complexities. Importantly, chromatographic performance remained virtually unaffected, with only a minor reduction in peptide retention. The mobile phase containing propionic acid was stable, instrument-compatible, and introduced a negligible background signal. Collectively, these findings challenge the long-standing reliance on formic acid and establish propionic acid as a robust, drop-in alternative for high-flow LC-MS workflows prioritizing MS sensitivity and proteome depth.

## Graphical abstract

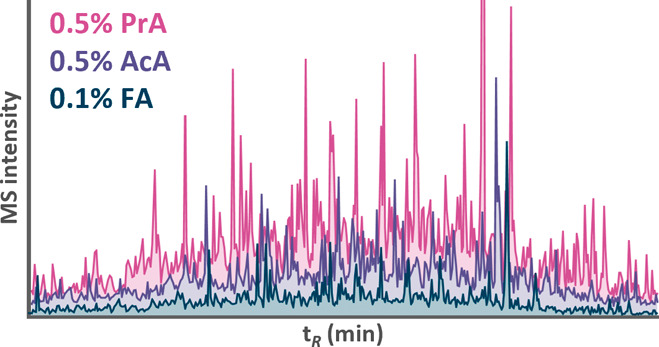

Reversed-phase liquid chromatography coupled with mass spectrometry (RPLC-MS) has become the gold standard in bottom-up proteomics.^1,2^ Several decades ago, a pivotal shift occurred in selecting acidic agents: formic acid (FA) replaced trifluoroacetic acid (TFA) as the default mobile phase additive for RPLC-MS workflows.^3–5^ Trifluoroacetic acid, a strong acid with a p*K*_a_ of 0.23, was initially favored for its dual benefits in RPLC.^6,7^ It maintains most residual silanol groups on silica-based stationary phases in the undissociated state, thereby minimizing unwanted electrostatic interactions. Additionally, its conjugate base, the trifluoroacetate anion, readily couples with protonated peptides and forms stable ion pairs with a reduced net charge, which have lower affinity to dissociated silanol groups and increased retention. These phenomena result in excellent chromatographic performance. However, while ideal for LC-UV analyses, TFA proved to be poorly compatible with electrospray ionization.^8,9^ The ion-pairing mechanism effectively “neutralizes” protonated peptides, shielding them from the electric fields transmitting ions into the ion optics, thus dramatically reducing MS signal intensity.^10^ In contrast, formic acid, with a p*K*_a_ of 3.75 and typically used at a concentration of 0.1%, generates almost 6-fold lower ionic strength in the mobile phase. While this results in weaker ion pairing and less efficient prevention of silanol interactions, it significantly enhances ESI efficiency, leading to higher MS signal intensity. Although the lower ion-pairing capacity and slightly higher pH of FA should theoretically compromise chromatographic performance compared to TFA, this effect is strongly dependent on stationary phase chemistry and peptide properties, and modern RPLC stationary phases have largely mitigated this issue.^11,12^ Innovations such as end-capping, steric shielding, or introducing positively charged groups into a stationary phase surface minimize silanol-related interactions, providing high separation performance even using mobile phases with reduced ionic strength. Our recent study demonstrated that the latter technology allows columns to maintain the separation performance even using mere 0.01% FA, further increasing MS sensitivity through reduced ionic strength.^13,14^ This mechanism is supposedly applicable to a structurally similar acid, acetic acid (AcA), with a p*K*_a_ of 4.76. At higher concentrations, typically around 0.5%, AcA provides acidity comparable to that of 0.1% FA while maintaining about half the ionic strength. It also reduces the surface tension of the mobile phase and concentrates in ESI droplets for a longer period due to its lower volatility, which further enhances the surface tension-reducing effect. Recent works by Lenčo et al. and Battellino et al. have demonstrated that AcA can significantly improve the number of peptide identifications by increasing MS signal intensity more than 2-fold on average,^15,16^ despite contradictory early reports.^5,17,18^ These studies indicate that AcA can offer a compelling alternative to FA in bottom-up proteomic workflows, particularly when prioritizing MS sensitivity.

Propionic acid (PrA), a homologous carboxylic acid, has been largely overlooked as a potential additive to the mobile phase. It has mainly attracted attention as a postcolumn additive that enhances MS signal intensity by modifying the composition of eluent droplets containing TFA.^9,10^ In our laboratory, PrA is frequently used as a dopant in the desolvation gas to improve ESI efficiency.^19–21^ Its performance inspired us to investigate the direct use of PrA in the mobile phase for LC-MS proteomics. To our knowledge, PrA has been applied only in a single RPLC-UV study investigating peptide retention and as an additive to a TFA-containing mobile phase in HILIC-MS for the analysis of basic drugs.^22,23^ With a p*K*_a_ of 4.88, 0.5% PrA produces a similar pH to that achieved with FA or AcA, but generates a lower anion concentration, suggesting additional potential for minimizing signal suppression. Moreover, its longer alkyl chain confers lower surface tension and volatility,^24^ properties expected to promote droplet formation and enhance ionization efficiency. For further mechanistic discussion, we refer readers to the section “Theoretical Considerations”.

In this study, we hypothesized that 0.5% PrA can outperform 0.5% AcA and 0.1% FA in MS sensitivity as an acidic additive to the mobile phase for RPLC-MS bottom-up proteomics. We compared its impact on ionization efficiency, separation performance, retention, and performance in peptide identifications to established setups using FA and AcA. The experiments were conducted independently at four research facilities, following local expertise and without constraints imposed by the principal investigators. We examined different column chemistries, including positively charged C_18_-, traditional C_18_-, and polyphenyl-bonded stationary phases, and evaluated performance for the samples of various complexity using a range of peptide sample loads. Recognizing the diversity of experimental setups in proteomics, our study encompassed analytical-, micro- (collectively referred to as high-flow), and nanoflow regimes, as well as MS instruments from two leading vendors, employing both standard and nanoESI sources, and both DDA and DIA acquisition modes. Additionally, we addressed the practical aspects of routine PrA use, including mobile phase stability and instrument compatibility, which was evaluated by GC-MS and ICP-MS analysis of leachables from the LC system. The findings presented here explore the utility of PrA as an alternative eluent additive for proteomic analyses requiring maximum sensitivity and extend prior investigations into AcA,^15,25^ particularly concerning separation performance and in-column artificial modifications.

## Experimental Section

### Chemicals and Reagents

Unless otherwise stated, chemicals, reagents, LC-MS solvents, and additives to mobile phases were purchased from Merck/Sigma-Aldrich, VWR, or Thermo Fisher Scientific in the highest available grade. Propionic acid was obtained in the LC-MS grade from Honeywell (Cat. No.: BJ49916-50ML) and the p.a. grade from Merck/Sigma-Aldrich (Cat. No.: 81910-1L). TFA was purchased from Honeywell. n-dodecyl-β-D-maltoside was obtained from Anatrace. One M Tris-HCl buffer, pH 7.5, was purchased from SERVA (Germany). Ethyl acetate was procured from Lach-ner, s.r.o. (Czech Republic). Peptides from the iRT and Alberta sets were synthesized by Royobiotech (China). Unused leftovers of freshly reconstituted bevacizumab (Avastin, Roche) were received from Multiscan Pharma (Czech Republic). The lyophilized Pierce HeLa Protein Digest Standard was purchased from Thermo Fisher Scientific. K562 cancer cell line digest standard was obtained from Promega. Sample preparation and the composition of the iRT and Alberta peptides mixture are described in the Supporting Information (Note S1).

### Instruments

Analytical- and microflow LC-MS analyses at the Faculty of Pharmacy in Hradec Králové (FPh) were performed using a Vanquish Horizon UHPLC system coupled to a Q Exactive HF-X mass spectrometer (Thermo Fisher Scientific) operating in positive ion mode with electrospray ionization via a HESI-II probe. At the Biomedical Research Centre at University Hospital Hradec Králové (BRC), micro- and nanoflow analyses were performed using a Dionex Ultimate 3000 UHPLC system hyphenated to a Q Exactive Plus mass spectrometer with a HESI-II probe and a Dionex Ultimate 3000 RSLCnano system coupled to an Orbitrap Exploris 480 mass spectrometer equipped with a NanoSpray Flex NG ion source and a FAIMS Pro Duo interface, respectively (Thermo Fisher Scientific). Experiments at the Organ Pathobiology and Therapeutics Institute, University of Pittsburgh (OPTIn) employed a nanoflow Evosep One LC system (Evosep Biosystems) interfaced with a timsTOF Ultra 2 mass spectrometer (Bruker Daltonics). Analyses at the Institute of Microbiology of the Czech Academy of Sciences in Prague (IMB) were conducted using a nanoflow Evosep One LC system coupled to a timsTOF SCP mass spectrometer (Bruker Daltonics). Detailed ion source and mass analyzer settings are specified in the Supporting Information (Tables S1 and S2). Unless otherwise stated, analyses were performed in triplicate. The LC-MS files from FPh, BRC, and IMB were deposited in the ProteomeXchange repositories via PRIDE with the identifiers PXD069554 and PXD070747 , and from OPTIn via MassIVE with the identifier MSV000099496.^26^

LC-UV determination of acidic additives in incubated samples of mobile phase was performed on a UltiMate 3000 RSLC system (Dionex) equipped with a diode array detector DAD-3000 RS and a 2.5 μL flow cell. The 54 elements, including heavy metals, released during eluent circulation were quantified using an Agilent 7900 ICP-MS system equipped with a collision cell ORS4. GC-MS profiling of volatile residues was conducted using an Agilent 7890 A system interfaced with an Agilent 5975 inert mass spectrometer operating in EI mode at 70 eV (Agilent Technologies).

### Effects of Propionic Acid on Analyses of Model Peptides

One microliter of the iRT and Alberta peptides mixture was separated using a 2.1 × 150 mm Acquity Premier column packed with 1.7 μm CSH C_18_ 130 Å particles (Waters) at 300 μL/min using a 10 min linear gradient of 0.5–30.5% B at 60 °C. Mobile phase components A and B were water and acetonitrile (ACN), respectively, acidified with 0.1% FA, 0.5% AcA, or 0.5% PrA. The MS spectra were evaluated in Skyline v22.1.^27^ Peptide isoelectric points were predicted using Isoelectric Point Calculator v2.0.^28^

### Effects of Propionic Acid on Peptide Mapping of Monoclonal Antibody

Tryptic peptides of bevacizumab were separated using the 2.1 × 150 mm Acquity Premier CSH C_18_ 1.7 μm column maintained at 60 °C using a 20 min linear gradient of 0.5–34.5% B and a flow rate of 250 μL/min. The mobile phase was acidified with FA, AcA, or PrA. Samples containing 5, 10, 50, 100, 500, and 1000 ng of the digest were injected twice. Five micrograms of peptides were also separated using a column temperature of 80 °C and a 90 min gradient of 0.5–32% B. The LC-MS data were searched as described in Note S2. Correlation analyses and linear regressions were performed in GraphPad Prism v10.4.1. Peptide hydrophobicity values were obtained using the Peptide Analyzing Tool (Thermo Fisher Scientific).

### Effects of Propionic Acid on Analytical- and Microflow Analyses of Complex Samples

At FPh, peptides from the Jurkat cell digest were separated using the 1.0 × 150 mm Acquity UPLC CSH C_18_ 1.7 μm column maintained at 60 °C using a 60 min linear gradient of 1.6–32% B and a mobile phase flow rate of 50 μL/min. The mobile phase was acidified with FA, AcA, or PrA. The injected peptide masses were 0.16, 0.5, 1.6, 5.0, and 16.0 μg. Five micrograms of peptides were also separated using a 120 min gradient of 1.6–30.4% B at 80 °C. Separations at 60 °C were repeated using five additional 150 mm columns, namely, 2.1 mm internal diameter (i.d.) Acquity Premier BEH C_18_ 130 Å 1.7 μm, BioResolve RP mAb Polyphenyl 450 Å 2.7 μm,^29^ bioZen Peptide PS-C_18_ 100 Å 1.6 μm, 1.5 mm i.d. HALO 160 Å ES-C_18_ 2.7 μm,^30^ and 1.0 mm i.d. Acclaim PepMap RSLC C_18_ 100 Å 2.0 μm. The peptide inputs were 3 μg for the 2.1 and 1.5 mm i.d. columns and 1.6 μg for the 1.0 mm i.d. column. The flow rate was 250 μL/min for the 2.1 mm i.d. columns, 125 μL/min for the 1.5 mm i.d. column, and 50 μL/min for the 1.0 mm i.d. column. The gradient method for each column was adjusted to ensure that most peptides eluted before the cleanup step, while remaining constant regardless of the mobile phase additive used. To smooth the pressure profile, a mixture of ACN/water (80:20, v/v) was used as component B of the mobile phase when operating at flow rates of ≤125 μL/min, with accordingly adjusted gradients. Analyses of 0.5 μg of peptides were replicated on a 1.0 mm i.d. HALO 160 Å ES-C_18_ 2.7 μm column using 0.1% FA, 0.01% FA, and 0.5% PrA. Analyses of 0.5 and 1.6 μg of peptides on the 1.0 mm i.d. Acquity UPLC CSH C_18_ 1.7 μm column were also performed using a mobile phase containing simultaneously 0.5% PrA and 3% dimethyl sulfoxide (DMSO) with a gradient of 0.5–31.2% B.^16,31^

Additionally, to assess the performance of acidic additives in a trap-elute configuration, 3 μg of peptides were injected into a system including a 2-position/6-port valve situated between the autosampler and the 2.1 × 150 mm Acquity Premier CSH C_18_ 1.7 μm column. The valve directed the flow through either a 2.1 × 5 mm Acquity UPLC VanGuard precolumn (later referred to as a trap column) packed with an identical stationary phase or a bypass capillary. Peptides were loaded into the trap column, and nonretained species were flushed for 2 min with 0.5% component B into the separation column. At 2 min, the valve was switched to the bypass line, preserving peptides retained in the trap column while a 30 min gradient of 0.5–40% B was applied to the separation column. Peptides remaining in the trap column were analyzed in the subsequent blank injection. The analysis involving the peptide loading into the separation column via the bypass capillary served as a control. Both columns were maintained at 30 °C.

To confirm the microflow LC-MS results, 0.5 μg of peptides from the HeLa cell digest dissolved in 0.1% FA were separated at BRC using the 1.0 × 250 mm HALO 160 Å ES-C_18_ 2.7 μm column maintained at 60 °C using a 60 min linear gradient of 1.6–37% B. A mobile phase acidified with AcA or PrA was used at a flow rate of 68 μL/min.

### Effects of Propionic Acid on Nanoflow Analyses of Complex Samples

At BRC, 0.25, 0.5, 1.5, and 5 ng of peptides from the HeLa cell digest were separated in four replicates using the 0.075 × 250 mm column packed with CSH C_18_ 1.7 μm 130Å particles maintained at 60 °C using a 60 min segmented gradient of 1.6–27.6% B in 54 min and 27.6–36% B in 6 min. A mobile phase acidified with FA, AcA, or PrA was used at a flow rate of 0.25 μL/min. The sample was injected directly using a filled 1 μL sample loop.

At OPTIn, 0.2 and 2 ng of peptides from the K562 cancer cell line digest standard reconstituted in 0.1% FA and 0.015% n-dodecyl-β-D-maltoside were separated using the 0.075 × 100 mm PepSep C_18_ 1.9 μm column (Bruker Daltonics). The column was maintained at 50 °C. The sample was loaded onto the separation column using C_18_ solid-phase extraction tips (EvoTips, EvoSep Biosystems) according to the manufacturer’s protocol, with peptide binding and washing performed in 0.1% FA. Peptides were separated using the 80SPD Whisper Zoom 16.3 min gradient method in 12 replicates. A mobile phase acidified with FA, AcA, or PrA was used at a flow rate of 0.2 μL/min.

At IMB, 0.04, 0.2, 0.4, and 2 ng of peptides from the HeLa cell digest desalted using EvoTips were separated in five replicates using the 0.075 × 50 mm Aurora Rapid-75 C_18_ 1.7 μm column (IonOpticks) maintained at 50 °C using the 80SPD Whisper Zoom and three mobile phase additives.

### Instrument Compatibility and Mobile Phase Stability

A volume of 250 mL of 0.1% FA and 0.5% PrA in water/ACN (1:1, v/v) was prepared in extra-clean inert Nalgene fluorinated ethylene propylene (FEP) bottles and was allowed to circulate in the Agilent 1260 Infinity II system for 7 days at a flow rate of 2 mL/min at room temperature. The flow path consisted of plastic tubing connecting the bottles to the pump, autosampler, stainless steel Viper capillaries, inlet filters, Viper unions, fused silica, and PEEK capillaries. The outlet of the last PEEK capillary was reinserted into the mobile phase containers. Within the experiment, the autosampler executed 200 injections of 20 μL of the same mobile phase. A control sample was stored in a sealed FEP bottle. Additionally, 400 mL of mobile phase was prepared in sealed 1 L InfinityLab borosilicate glass bottles (Agilent Technologies) and stirred at 400 rpm for 7 days at room temperature. The elements released during circulation were quantified using ICP-MS as described elsewhere.^32^ The ESI-MS profile was examined using direct infusion into the Q Exactive HF-X mass spectrometer at 25 μL/min. Volatile residues were profiled with GC-MS using an optimized US EPA Method 8270 as described in Note S3.^33^

A volume of 300 mL of 0.1% FA, 0.5% AcA, and 0.5% PrA was incubated at 20 °C for 7 days in 500 mL amber Duran glass bottles with plastic caps accommodating a plug and a mobile phase safety filter. At six time points, mobile phases were sampled, and 1 μL was injected into a 2.1 × 150 mm Acclaim Organic Acid 120 Å, 3 μm column maintained at 30 °C.^34^ The concentration of acids was determined using an isocratic elution with 100 mM sodium sulfate in 0.1% TFA. The flow rate was 210 μL/min. Data were acquired at a wavelength of 214 nm and a sampling frequency of 20 Hz. In parallel, the acidity of mobile phases was monitored using a Hanna Instruments pH 212 potentiometer equipped with a combined glass and silver/silver chloride electrode.

## Results and Discussion

### Theoretical Considerations

Electrospray ionization efficiency is influenced by the mobile phase acidic additive, which defines the ionic strength, surface tension, and volatility of the mobile phase. These parameters govern the formation and behavior of charged droplets, desolvation kinetics, and ion release, which are crucial for achieving high sensitivity in LC-MS bottom-up analyses.

A minimal ionic strength provided by dissociating additives is essential to ensure stable spray formation and efficient ion generation.^35,36^ This requirement stems from its influence on the solution’s electrical conductivity *κ*, which defines the spray current *I* during Taylor cone formation:^37^

1 $$I\propto \sqrt{\frac{\gamma \kappa Q}{\epsilon_{0}}}$$

where γ is surface tension, *Q* is the mobile phase flow rate, and *ϵ*_0_ is the vacuum permittivity.^37–39^ Without sufficient ionic strength, *κ* is too low to sustain the electrospray current to accumulate enough charge at the liquid surface, resulting in intermittent cone formation, poor droplet charging, and low analyte ion yield.^40^ Acidic additives also serve as essential proton donors for peptide amino groups during ESI. However, beyond a certain concentration threshold, an additive begins to compete for ionization with analytes, lowering their ion yield.^36,41^ Empirical models by *Kebarle and Tang* and *Enke* quantify the ionization process as the partitioning of a finite pool of charges between species in the droplet:

2 $$I_{A}=\frac{c_{A}\alpha_{A}}{c_{A}\alpha_{A}+\underset{i\neq A}{\sum }c_{i}\alpha_{i}}$$

where *I*_*A*_ is the ion intensity of the analyte, *c*_*A*_ and *c*_*i*_ are the concentrations of the analyte and other ionic species, and α_*A*_ and α_*i*_ are their empirical response factors explaining individual ion charging efficiencies.^40,42^ The pool of available charges is given by the spray current and Rayleigh limit.^43^ Upon increasing the concentration of a competing additive cation, the fraction of charge allocated to the analyte decreases, thereby lowering its ion intensity. Additionally, the anions of strong acids form stable ion pairs with protonated groups of peptides that suppress ionization.^8,9^ As the additive changes from 0.1% TFA to 0.5% PrA, the ionic strength decreases inversely with p*K*_a_, reducing ion-pairing interactions and ionization competition, thereby positioning PrA as the most promising additive for maximizing ionization efficiency (Table 1).

**Table 1:** Physicochemical Properties of Acidic Eluent Additives

**Pure acid**	TFA	FA	AcA	PrA
p*K*_a_	0.23	3.75	4.76	4.88
Surface tension at 25 °C, mN/m^44,45^	13.5	37.0	27.1	26.2
Boiling point, °C	72.4	100.7	118.1	141.2
Vapor pressure at 20 °C, mmHg^46^	82.5	34.5	11.2	2.9

Solution (v/v)	0.1% TFA	0.1% FA	0.01% FA	0.5% AcA	0.5% PrA
pH	1.9	2.7	3.2	2.9	3.0
Molarity, mM	13.1	26.5	2.6	87.4	67.0
Anion concentration, mM	12.8	2.1	0.5	1.2	0.9

In the series of carboxylic acids, the surface tension decreases with increasing alkyl chain length. Its impact on the resulting mobile phase surface tension is further strengthened by the greater volume concentrations of 0.5% compared to 0.1%. Surface tension regulates the balance between cohesive forces and electrostatic repulsion in charged droplets.^38,40^ A central mechanistic constraint is the Rayleigh limit, which describes the maximum charge *q* a droplet of a radius *R* can carry before undergoing Coulomb fission:

3 $$q=8\pi R\sqrt{\epsilon_{0}\gamma R}$$

Mechanistic interpretations based on Rayleigh’s theory predict that as the charge approaches the limit, electrostatic repulsion overcomes the cohesive force from surface tension, leading to droplet fragmentation. Lowering surface tension reduces droplet charge capacity, making it more likely to undergo Coulomb fission at larger sizes or lower total charges.^43,47^ Reducing surface tension also decreases the critical electric field strength *E*_*onset*_ required to initiate Taylor cone formation from a capillary of radius *r*, creating finer droplets at a constant voltage or enabling stable electrospray at lower voltages:^39^

4 $$E_{onset}∼\sqrt{\frac{2\gamma }{\epsilon_{0}r}}$$

In practical terms, solvents with reduced surface tension promote more frequent droplet fission into smaller progeny droplets, which in turn accelerates desolvation and enhances ion liberation.^39,40^ Such a consequence is proposed when replacing FA with AcA, and especially with PrA.

In contrast to surface tension, AcA and PrA exhibit lower volatility than FA, which may initially seem a drawback. Applied to mobile phase solvents, lower volatility indeed results in slower desolvation and poorer ion yield. However, we hypothesize that less volatile additives tend to concentrate in the droplets, thereby boosting their positive effect on surface tension and serving as a longer-lasting proton donor. The effect on the surface tension is similar in principle to that proposed for supercharging agents, such as *m*-nitrobenzyl alcohol, which enhance ionization by persisting in evaporating droplets and promoting further droplet fission.^48,49^ The proximity of the boiling points of *m*-nitrobenzyl alcohol and PrA (141 vs 175 °C) supports the proposed mechanism.

Apart from ESI enhancement, we proposed that the lower acidity of PrA may reduce in-column peptide modifications induced by exposure to low pH and elevated column temperature. They include the cyclization of N-terminal Glu and Gln, dehydration of Asp, oxidation of Met, and nonenzymatic cleavage at Asp.^29^ Previous efforts to reduce artificial modification by elevating pH using a lower concentration of FA had surprisingly resulted in slightly higher abundance of identified modifications.^13^ That was exclusively attributed to increased MS sensitivity since modified peptides are typically low-abundant and easily fall below the intensity threshold needed for identification in DDA experiments. In this study, this phenomenon is extensively investigated at both the levels of quantities of individual modified-parent peptide pairs and the abundances of modified peptides among the total identified peptides.

### Effects of Propionic Acid on Analyses of Model Peptides

The effect of PrA on ESI and chromatographic behavior was initially investigated using a simple peptide mixture separated on an analytical-bore column packed with a C_18_-bonded stationary phase bearing a positively charged surface. iRT peptides are well-characterized standards used for retention time recalibration.^50^ Four Alberta peptides are acetylated at their N-termini and contain 1 to 4 lysines protonated at acidic pH.^4^ All 11 peptides exhibited higher MS signal intensity with PrA than FA, while only eight were more intense using AcA. On average, total peak intensities using PrA were higher than AcA by 28.2 ± 7.3% (Figure 1). Although mostly positive, the intensity changes were highly variable among different peptides, indicating a composition-dependent effect of an additive. For Alberta peptides, the signal enhancement gradually declined with increasing net charge. The two least acidic iRT peptides exhibited the lowest increase in intensity. Altogether, it aligns with the previously observed favoring of acidic peptides by AcA-based eluents.^15^ PrA demonstrated a similar trend, showing a substantial increase in intensity for three of four Alberta peptides, whereas for AcA, this was observed only for the first, most acidic analyte.

**Figure 1:**
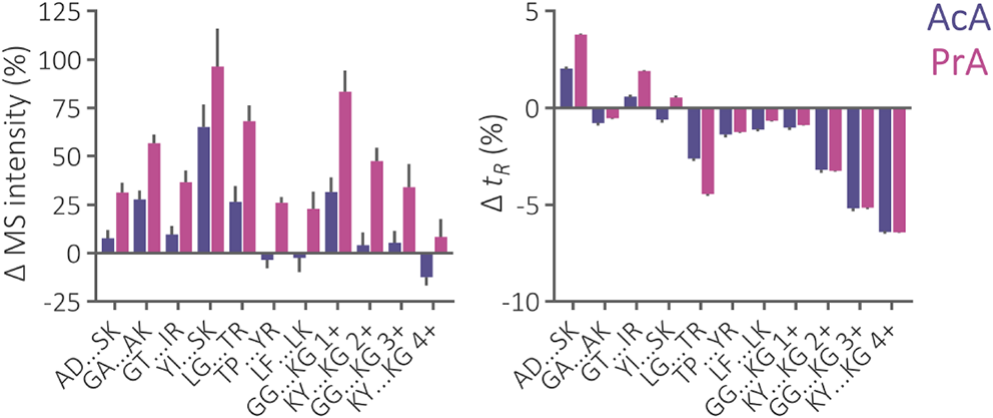
Relative change of total precursor MS intensity and retention time (*t_R_*) of peptides from iRT and Alberta sets separated using a 2.1 × 150 mm Acquity Premier CSH C_18_ column and mobile phase containing 0.5% AcA or 0.5% PrA in comparison to the separation using 0.1% FA. The iRT peptides are listed from left to right in the order of increasing isoelectric point. The number of protonated amino groups is indicated for Alberta peptides. For iRT and Alberta peptide properties, see Figure S1.

Changes in separation selectivity showed a similar pI-dependent trend, with retention times decreasing as peptide basicity increased (Figure 1), again aligning with the previous findings.^13,15^ The more positively charged groups the peptide has, the more sensitive its retention is upon reducing the ionic strength.^13,51^ Reduced retention results from the weaker ion-pairing properties of AcA and PrA, which are responsible for increasing peptide hydrophobicity in the case of strong acids, such as TFA. The peptide peaks tended to broaden, with marginally worse results for PrA than for AcA (Figure S1). Nevertheless, no peptides showed a greater *w*_0.5_ increase than 10%, and enhanced ionization has greatly outweighed the negative impact of peak broadening on the chromatographic peak height.

We also observed that AcA and PrA increase the abundance of multiply charged precursors, particularly for the Alberta peptides that contain many chargeable moieties (Figure S1). Maximum MS sensitivity is achieved when a peptide carries a single charge state, allowing for MS2 dissociation of a single dominant precursor ion. Therefore, we again compared peak heights, focusing on the most abundant precursor (Figure S1). Only the last Alberta peptide was noticeably affected by the comparison method, while the base peak intensities of all other peptides increased almost proportionally to their total precursor intensities.

### Effects of Propionic Acid on Peptide Mapping of Monoclonal Antibody

Tryptic digests of monoclonal antibodies typically yield a few dozen unique peptides, enabling statistically robust analysis of strong population-level dependencies. At the same time, they provide high individual peptide concentrations, ensuring consistent surpassing of DDA intensity thresholds, which facilitates the detection of low-abundance chemical modifications and multiple peptide precursors. Therefore, bevacizumab peptides were separated at multiple injected mass loads using a column with the same analytical i.d. and positively charged C_18_ chemistry as those used for iRT and Alberta peptides. AcA and PrA increased the peak area of individual peptides by 53% and 113% on average, resulting in a greater number of peptide identifications in analyses of all sample quantities (Figure 2).

**Figure 2:**
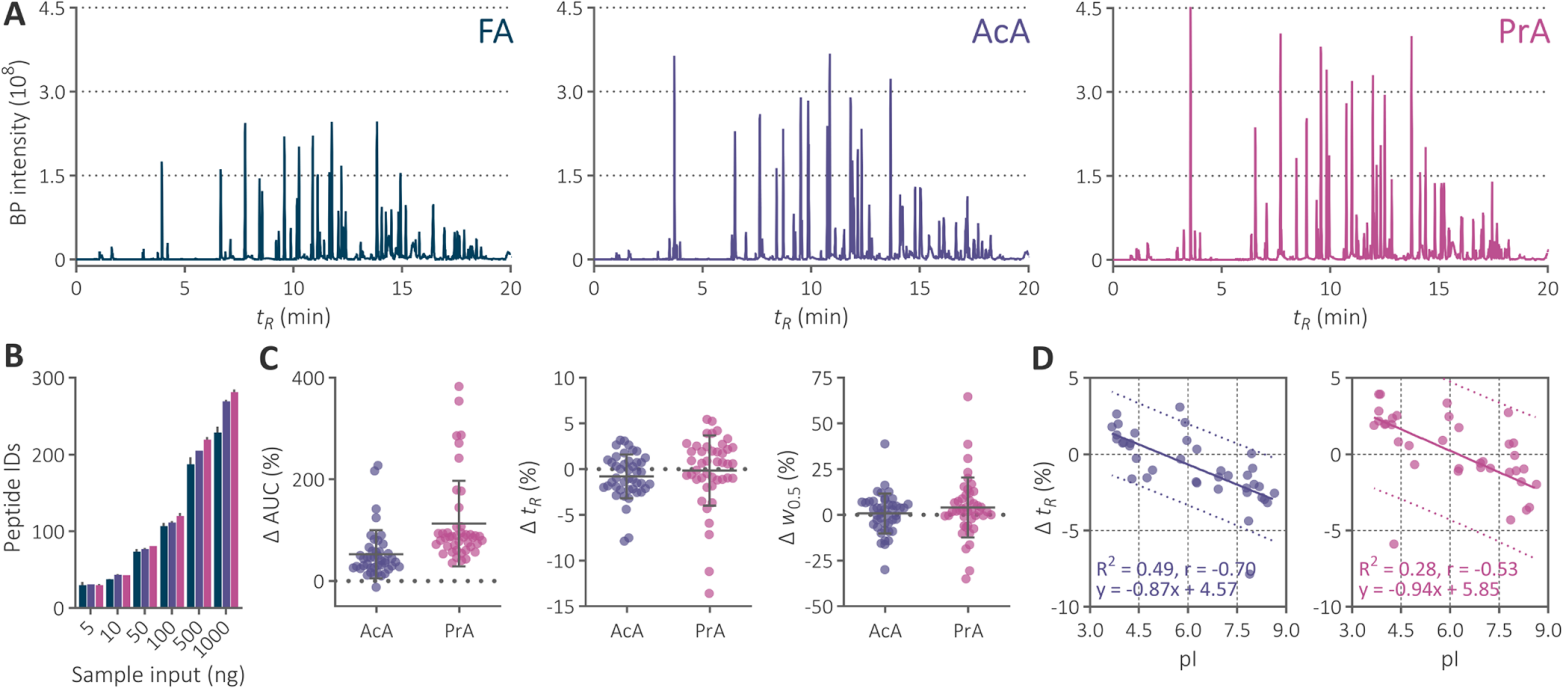
(A) Base peak chromatograms of 1 μg of bevacizumab peptides separated within a 20 min gradient using the 2.1 × 150 mm Acquity Premier CSH C_18_ column maintained at 60 °C and mobile phase containing FA, AcA, or PrA. (B) Total identified peptides in analyses of five sample inputs using three additives. Database search was performed with semitryptic specificity, allowing up to two missed cleavages. (C) Distribution of relative change of peak area (AUC), *t_R_*, and peak width at half height (*w*_0.5_) of 44 representative peptides normalized to those observed using FA-containing mobile phase. The peak areas of all identified precursors were summed. The mean and standard deviation from duplicates are illustrated. (D) Dependence of *t_R_* change on peptide isoelectric point (pI) when switching to AcA and PrA from FA with linear regressions. The equations of the linear regression, determination coefficients, and Pearson correlation coefficients are shown below. The retention times of 38 unmodified peptides were evaluated. Colored dots illustrate 90% prediction bands.

With a higher sample complexity, we observed an average *t*_*R*_ decrease of 0.8% using AcA and 0.1% using PrA, compared to FA (Figure 2). Deviations in *t*_*R*_ were primarily driven by peptide acid–base properties, as indicated by Pearson correlation coefficients of r = −0.70 (*p* < 0.0001) and r = −0.53 (p = 0.0007) for AcA and PrA, respectively, showing a *t*_*R*_ decrease with increasing pI. Switching to PrA led to an average *w*_0.5_ increase of 4.1%, while AcA broadened peaks by only 0.9% (Figure 2). Both results are generally negligible when considering the advantages of alternative acidic additives in ESI enhancement. The more efficient generation of multiply charged precursors followed the same trend as for the model Alberta peptides (Figure S2).

Monitoring the common modification sites in the bevacizumab structure, we found that the relative quantities of modified peptides increased slightly upon replacing FA with AcA and PrA, even with a short 20 min separation method and a moderately elevated column temperature of 60 °C (Figure 3). An extended 90 min gradient separation at 80 °C corroborated our observations. Together with the previously observed increase in artificial modifications at a lower FA concentration of 0.01%,^13^ these results indicate that elevating the mobile phase pH adversely affects the abundance of commonly monitored modifications. The increase in artifact levels cannot be attributed to improved MS sensitivity, as it would proportionally increase the signals of both modified and unmodified peptides, leaving their AUC ratio unchanged. Therefore, maintaining pH at 2.7 using 0.1% FA and avoiding elevated column temperature appears to be the most efficient ways to prevent artificial modifications.

**Figure 3:**
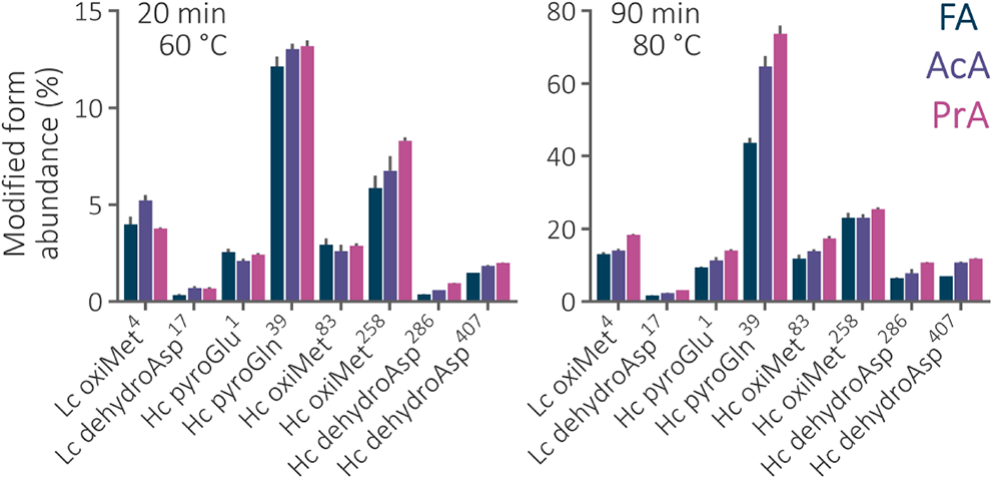
Relative abundance of the modified peptide forms in the 20 and 90 min separations of bevacizumab peptides using the 2.1 × 150 mm Acquity Premier CSH C_18_ column maintained at 60 and 80 °C. The abundance was calculated as the peak area of all the precursors of the modified peptide divided by the summed area of both peptide forms. The most abundant modified peptide containing the modified amino acid was used. Abbreviations: *Lc* – light chain, *Hc* – heavy chain. The superscripted numbers correspond to the position of the modified amino acid in the chain sequence.

### Effects of Propionic Acid on Analytical- and Microflow Analyses of Complex Samples

To refine and validate our findings, we compared acidic additives using separations of complex digests of human cell lysates. Such a large data set provides an accurate means for comparing peptide identifications, which is the ultimate output of proteomics analysis that reflects MS sensitivity. Jurkat cell protein digests were separated using columns of various i.d. packed with positively charged C_18_-, traditional C_18_-, and polyphenyl-bonded stationary phases. Regardless of peptide sample load or column used, we observed a significant increase in peptide identifications by both AcA and PrA (Figure 4), with PrA showing robust superiority over AcA by an average of 11.7% ± 8.1%. Microflow analyses of digested HeLa proteins at BRC showed a 6.0% ± 1.0% increase. The AUC increase correlated with peptide acidity, aligning with the findings of Battellino et al. regarding AcA (Figure S3). Apart from acidity, we found a positive correlation between signal increase and peptide hydrophilicity. The relative increase in identifications due to the additive switch declined naturally with increasing peptide sample load because of gradual saturation of the MS2 capacity under fixed DDA settings and chromatographic gradient length.^52^

**Figure 4:**
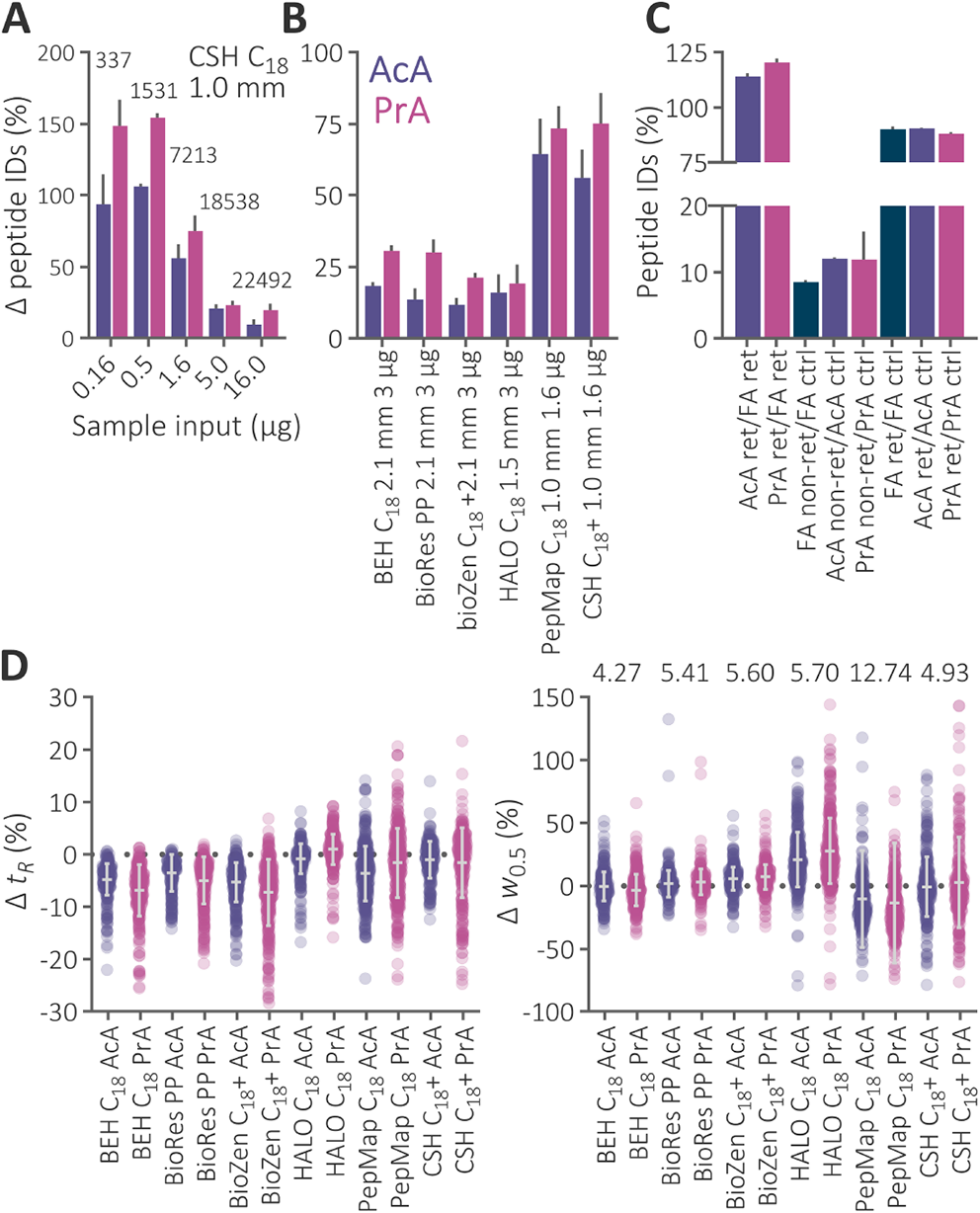
(A) Relative increase in the number of identified peptides from various sample inputs of digested Jurkat cell proteins separated within a 60 min gradient using the 1.0 × 150 mm Acquity UPLC CSH C_18_ column maintained at 60 °C and mobile phases containing AcA and PrA in comparison to FA. An average number of peptides identified under the FA conditions is highlighted above. (B) Relative increase in identifications in analyses using different columns. Data set descriptions include stationary phase ligand, internal column diameter, and sample load. A plus sign indicates a positively charged surface of the stationary phase. (C) Ratios of peptide identifications in the experiment involving the trap-elute configuration. Abbreviations: *ret* – fraction of peptides retained in the trap column during a 2 min isocratic loading step and analyzed in the subsequent blank injection, *nonret* – fraction of peptides that eluted from the trap column during the loading step, and *ctrl* – number of identifications using direct injection of peptides into the separation column through a bypass capillary. (D) Relative change of *t_R_* and *w*_0.5_ of 400 peptides from digested Jurkat cell proteins analyzed on different columns using mobile phases containing AcA and PrA relative to FA. The mean and standard deviation are illustrated. Peptides were randomly selected from each column data set separately. The spectra were manually revised to ensure correct peak selection and integration. The numbers above correspond to average *w*_0.5_ (s) in the FA data sets.

A sample input of 16 μg yielded 77% of the maximum number of identifications theoretically achievable with the FA-containing mobile phase in a single analysis, as predicted by the saturation curve model.^52^ Even at this high sample load, AcA and PrA preserved the ability to further increase the number of identified peptides by 9.6% and 19.8%, respectively. However, this nonlinear saturation behavior prevents a direct comparison of the increase between columns with different internal diameters. Different separation performance of columns also biases such a comparison. That is why varying identification outputs from individual columns using alternative additives are barely explainable.

During analyses on different columns, we also compared the performance of PrA with that of the recently explored 0.01% FA. Despite maintaining a higher ionic strength than 0.01% FA (Table 1), PrA provided 46.5% more identified peptides than 0.1% FA, while 0.01% FA increased identifications by only 28.4%, demonstrating that other factors, such as surface tension and volatility of the additive, contribute to ESI efficiency. We next examined the combination of PrA with DMSO, which enhances ionization efficiency and/or reduces the number of peptide charge states.^53^ The primary aim was to reveal potential synergism or antagonism in the effect on peptide signal intensities, as it was discovered for AcA.^16^ Compared to PrA, we obtained 30.6% and 15.4% additional identifications from the combination of PrA and 3% DMSO using 0.5 and 1.6 μg of injected peptide masses, respectively, with opposite effects of PrA and DMSO on charge state distribution (Figure S4). Furthermore, we expected that an additional boost in sensitivity could be achieved by reducing the PrA concentration, as observed for FA. However, the reduced ionic strength associated with low-concentration PrA led to increased peak broadening, which eventually outweighed the improved ionization efficiency, resulting in lower peak intensities in analyses of iRT and Alberta peptides using CSH and HALO columns. Therefore, maintaining the original PrA concentration is recommended to ensure consistently high chromatographic performance across a broad range of stationary phases.

In a set of randomly selected 400 peptides, the average *t*_*R*_ reduction on the CSH C_18_ column decreased to 1.0% using AcA and 1.6% using PrA (Figure 4). Together with other columns, the average *t*_*R*_ decrease was 3.2% and 3.5%, corresponding to 0.40% and 0.46% decrease in apparent content of ACN at elution, respectively, calculated from the linear gradient by converting retention times to the corresponding mobile phase composition. The relative decrease in retention positively correlated with peptide hydrophilicity (Figure S5). Ion-pairing agents exert the greatest retention-enhancing effect on the most hydrophilic peptides, acting as a key factor in ensuring at least minimal retention,^54^ particularly on low-retentivity columns, such as those bearing a positively charged surface. This accounts for the most pronounced retention-reducing effect observed upon lowering the ionic strength of the mobile phase for peptides with the weakest retention. This aspect may be critical for the proteomics analyses focused on the least hydrophobic peptides, where signal enhancement might be outbalanced by excessive loss of peptides in the trap-elute configurations.

To assess the extent of this loss, we simulated the trap-elute configuration using a trap and separation column packed with the same stationary phase, and quantified the fractions of retained and nonretained peptides with a direct injection into the separation column serving as a control (Figure 4). Although the fractions of nonretained peptides were virtually identical for AcA and PrA (around 12%) compared to FA (8.5%), the retained fraction using PrA slightly decreased to 88.2% compared to AcA and FA (both around 90%). Collectively, AcA preserved its 14% increase in identifications over FA, even when using the trap-elute configuration. In contrast, peptide loss during the trapping phase with PrA modestly reduced the number of extra identified peptides from 23.0% to 20.5%. This corresponds to the previously observed larger *t*_*R*_ decrease for hydrophilic peptides when using PrA compared to AcA (Figure S5). It also highlights the need to reassess the trade-offs of using PrA when targeting early eluting peptides, particularly in a trap-elute configuration. However, the loading solvent may still contain standard FA to compensate for this loss, as it was proposed for TFA when using FA in the separation mobile phase.^55^ Alternatively, the trap column can be packed with a polar-embedded stationary phase to improve retention of hydrophilic species.

Except for the data sets obtained using the HALO and BioResolve columns, previously observed dependence of *t*_*R*_ change on the peptide acid–base properties was confirmed (Figure S6). Greater slopes of linear regressions for PrA indicate higher sensitivity to peptide pI, albeit with lower prediction strength described by the determination coefficients. We believe that the fitness of models could be significantly improved by excluding peptides with the shortest *t*_*R*_, as they are influenced by the correlation mentioned above. While the lack of correlation between pI and *t*_*R*_ on the BioResolve column can be explained by a greater contribution of π-π interactions, the mechanism underlying the observations on the HALO column remains unclear to us.

The average peak broadening did not exceed 5% for both AcA and PrA (Figure 4). The exceptions were the HALO column, which showed a noticeably higher *w*_0.5_, and the PepMap column, which exhibited surprisingly improved peak shapes. However, even this extent of peak broadening did not outweigh the effect of increased ESI efficiency on identifying peptides using alternative additives (Figure 4). The peak broadening in the HALO column data sets positively correlated with *t*_*R*_ (Figure S7), but not in other column data sets. No other correlations of additive-induced peak broadening with peptide properties, such as pI and molecular weight, were found.

The previously observed increase in the rate of artificial modification at the level of individual parent-modified peptide pairs (Figure 3) was supported by a comparison of modified peptide abundances in large-scale identification data sets (Figure S8). Although the increase was only significant at substantially elevated column temperatures and extended separation methods, while remaining minor under standard conditions, we recommend continuing to use 0.1% FA in applications where in-column artificial modification must be minimized. These include research on the relationship between the mentioned post-translational modifications and biological processes, as well as the quantification of critical quality attributes in monoclonal antibody drug formulations that can be biased by the introduction of excessive modifications during sample analysis.^21^ On the other hand, we believe that the performance of common proteomics analyses would not be impacted by a 1–2% increase in the total abundance of artifacts that normally fluctuates around 5%.

### Effects of Propionic Acid on Nanoflow Analyses of Complex Samples

Although high-flow configurations have gained traction as advances in instrumentation have narrowed the difference in proteomics performance,^31,56,57^ most researchers continue to favor nanoflow LC-MS systems. Given the mechanistic differences between standard and nanoESI sources,^58–60^ we evaluated whether the sensitivity advantage of PrA over AcA and FA observed in high-flow experiments persists under nanoflow conditions using sample loads typical for single-cell proteomics. Except for the analyses performed at BRC, AcA and PrA produced comparable numbers of peptide identifications, both significantly higher than those with FA, especially at lower sample loads (Figure 5). These findings demonstrate that AcA and PrA should be preferred over FA for nanoLC analyses of samples with limited quantity.

**Figure 5:**
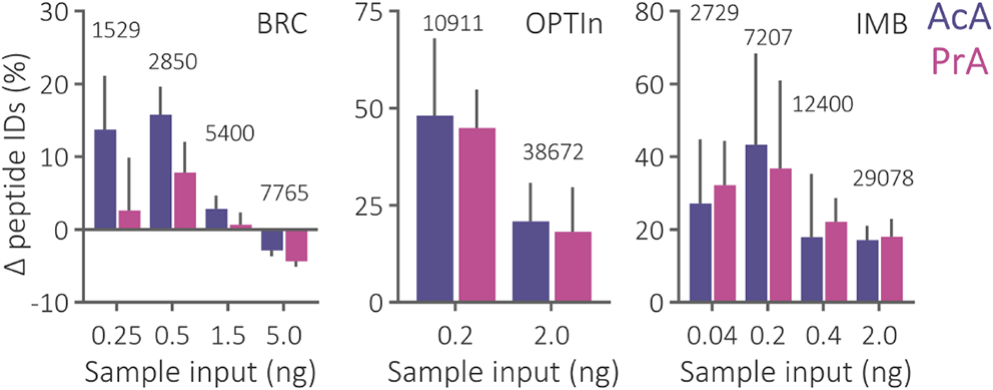
Relative change in the number of identified peptides in nanoflow analyses of various sample inputs separated using a mobile phase containing AcA or PrA in comparison to FA. An average number of peptides identified under the FA conditions is highlighted above. Results from three research facilities exploiting Orbitrap Exploris 480 (BRC), timsTOF Ultra 2 (OPTIn), and timsTOF SCP (IMB) mass spectrometers are illustrated.

Inspection of the peptide peaks unbroadened by the additive switch revealed marginal intensity differences between AcA and PrA. We attribute the similar efficiency of these additives in nanoESI to the inherently diminished influence of eluent surface tension under nanoflow conditions, as the very low flow rates produce much smaller initial droplets that rapidly reach the Rayleigh limit and undergo ionization without extensive solvent evaporation.^61,62^ Furthermore, nanoESI typically operates without heated nebulizing gas, which, in standard ESI, may accelerate evaporation of AcA relative to PrA due to its lower boiling point. The absence of this effect in nanoESI prevents the premature loss of AcA, acting as both a proton donor and a surface-active agent, resulting in comparable ionization efficiencies for both additives. Given the nearly identical ESI efficiency, the reduced performance of PrA relative to AcA in the results obtained from BRC was attributed to more pronounced peak broadening: the average *w*_0.5_ increased by 12.2% compared to FA, whereas AcA showed only a 5.2% increase. The same phenomenon likely led to fewer identifications using both additives in comparison to FA with increasing sample input. The cause of this peak broadening remains unclear to us. Although the column used was packed with the same CSH C_18_ particles as the 1.0 and 2.1 mm i.d. columns, which demonstrated negligible changes in *w*_0.5_, we acknowledge that multiple factors might contribute to this observation. For instance, the average *w*_0.5_ increase at OPTIn was below 5% for both acids.

### Instrument Compatibility and Mobile Phase Stability

In contrast to the analysis of small molecules, the composition of mobile phases in proteomics workflows is seldom reoptimized once established. This conservatism likely stems from the field’s reliance on empirically validated formulations and from concerns regarding unexplored effects on instrument performance or data quality. To address such concerns, we deemed it essential to assess the compatibility of a new additive with respect to instrument safety, mobile phase stability, and MS background noise. Formic acid, a default additive, is widely considered safe and fully compatible with routine operation. To our knowledge, no study has explicitly assessed the safety profile of AcA. Nevertheless, several proteomics groups have used AcA without reported complications,^15,63^ and its application is also widespread outside the proteomics field.^64,65^ Since PrA is a weaker acid than both FA and AcA, and a 0.5% solution yields a pH within the operational range of standard instrumentation, we did not expect its safety profile to be inferior.

In the LC-MS system, the mobile phase comes into contact with various components, including storage containers, chromatographic columns, tubing, capillaries, pump head metal components, and seals. These are typically made of glass, stainless steel, titanium or titanium-based alloys, fused silica, PEEK, and other organic polymers. Trace amounts of these materials may leach into the mobile phase and be carried into the mass spectrometer. Nonvolatile substances can accumulate in the MS front end, potentially causing contamination, while others may persist in mass spectra, elevating background noise. Quantifying the spectrum of compounds in the mobile phase that has passed through the entire LC system offers a practical way to evaluate leaching rates, thereby providing insight into the safety of the mobile phase.

To compare PrA to FA, we allowed the acidified 50% ACN to circulate incessantly through the LC instrument at a high flow rate for 7 days, while another portion of the sample was stored in a glass container. The exploited LC instrument had no polymeric lining that prevents the mobile phase from contacting metal surfaces. The flow path did not include the chromatographic column because of its ability to retain trace amounts of metals.^66^ We believe that reducing mobile phase acidity would only improve column lifetime, and searching for the opposite effect is unnecessary. The samples were quantified for 54 elements by ICP-MS, nontargeted direct infusion ESI-MS, and GC-MS (Table S3).

ICP-MS analysis revealed increased concentrations of Fe, Cr, Ni, Cu, Mo, and Mn, indicating the release of these elements from stainless steel components (Figure S9). With an increased iron concentration, we detected positively charged ions in ESI-MS spectra at *m*/*z* 548.94, 621.97, 696.01, and 770.04, corresponding to carboxylate oxygen-centered triangular complexes formed between iron and PrA.^67^ Analogous complex formation has been reported for AcA at *m*/*z* 538.96, which may suggest similar metal-leaching properties. A minor increase in abundance of (2ACN+Cu)^+^ ions with *m*/*z* of 144.98 and 146.98 was also observed along with the increased copper levels. The boron concentration remained unchanged, confirming that the mobile phase additives had no effect on the leaching of glass. The quantities of trace elements typical for glass, such as Na and K, fell below the lower limit of quantitation of 10 and 55 ppb, respectively, in all the samples. The limits of quantification for these metals are close to the common maximum concentrations allowed by LC-MS solvent manufacturers in their products (Table S3), so any increase in their concentrations due to switching to PrA would still not exceed these limits. An increased concentration of Co in samples stored in glass containers remained unexplained, as laboratory glass may contain only trace amounts of this metal. An aggregate mass of all the quantified elements leached by FA and PrA within the experiment was 48.6 ± 0.1 μg and 86.8 ± 1.3 μg, respectively, representing a 1.8-fold increase. Given that the samples completed almost 81 full circulation cycles through the LC instrument, an extrapolated amount of elements leached under normal operational conditions is insignificant. GC-MS profiling detected no anticipated contaminants. The concentrations of all mobile phase additives remained constant throughout the 7-day stability examination, with RSDs of <1% and <3% for the mobile phase additives and pH, respectively.

The intensity of the MS background noise of the mobile phases was comparable across additives and even decreased for AcA and PrA at lower flow rates (Figure S10). Metal-associated ion clusters in the range of 530–630 *m*/*z* were observed only in the AcA spectra, while the increased noise in the PrA spectra was primarily caused by ions below 350 *m*/*z*. In contrast to DMSO, the use of alternative additives had no long-term effects on the MS background.^52^ In addition, PrA did not exhibit any unpleasant odor during LC-MS operation. When mobile phases were prepared in a fume hood, the handling of PrA was odor-neutral. During the study, the LC-MS grade PrA from Honeywell was discontinued; therefore, starting with the analyses of bevacizumab peptides, we used the p.a. grade PrA (≥99.5% GC purity) from Merck/Sigma-Aldrich. Surprisingly, the p.a. grade product produced lower background noise (TIC 2.2 × 10^6^ vs 2.7 × 10^6^), which dispelled the purity-related concerns.

## Conclusion

Small organic acids are traditionally favored as acidic additives to the mobile phase in RPLC-MS bottom-up proteomics analyses, with formic acid long regarded as the gold standard. Recently, acetic acid has been revisited as a superior alternative that was largely abandoned decades ago, following early reports showing its advantages over FA. In this study, we broadened the scope of applicable acidic additives within the homologous series of carboxylic acids by introducing propionic acid. Acknowledging the general conservatism within the proteomics community, evidenced by the slow adoption of even clearly beneficial methodological advances, we systematically investigated a broad range of aspects related to PrA utilization.

Due to its lower ionic strength, surface tension, and volatility, 0.5% PrA significantly enhanced electrospray ionization efficiency and outperformed 0.5% AcA, 0.01% FA, and 0.1% FA in terms of MS sensitivity using analytical- and microflow configurations. This resulted in an average 12% increase in peptide identifications under PrA conditions compared to AcA, with the greatest benefit in AUC for acidic and hydrophilic peptides. In contrast, no improvement was observed in the nanoLC configuration, consistent with the comparable ionization efficiency of nanoESI sources using both additives. Similarly to AcA, an additional increase in identifications was achieved when it was combined with DMSO.

Chromatographic performance under PrA conditions remained comparable to that with FA, with peptide peak widths increasing by no more than 5% on average. Due to its relatively weaker ion-pairing properties, PrA induced a minor reduction in peptide retention, most notably affecting the hydrophilic species, in contrast to the greatest signal increase. The most pronounced retention decrease, previously observed for AcA in high-pI and weakly retained peptides, was also confirmed for PrA, with slightly higher significance. Therefore, PrA may not be the ideal additive for workflows focused on hydrophilic peptides, especially when using trap-elute configurations. Nevertheless, for most standard proteomics applications, PrA consistently outperformed other additives in terms of peptide identifications, even when used with columns that exhibited the most pronounced peak broadening and retention decrease.

The use of PrA in combination with elevated column temperatures should also be used with caution in studies of post-translational modifications that may also occur in the column and interfere with the modifications of interest formed before the analytical phase. Although we initially hypothesized that reducing mobile phase acidity would lower the abundance of artificial modifications, our results indicated that 0.1% FA remains the safest known additive in this context. Still, we believe that the observed increase in modifications under PrA conditions is unlikely to negatively impact standard proteomics experiments that are not explicitly focused on post-translational modifications.

Furthermore, mobile phases containing PrA were fully compatible with LC-MS instrumentation, did not increase MS background noise, and remained stable over standard storage durations. Taken together with its functional advantages, we conclude that adopting propionic acid represents a simple, low-cost, and powerful strategy to substantially enhance proteomic performance in high-flow LC-MS analyses, offering an attractive step before pursuing more extensive and expensive instrumental optimizations.

## Supplementary Material



## Notes

Sample preparation (Note S1); search parameters for bottom-up LC-MS data (Note S2); GC-MS profiling (Note S3); ion source settings (Table S1); settings of MS1 and DDA/DIA experiments (Table S2); concentration (ppb) of elements influenced by the selection of acidifier (Table S3); effects of alternative additives on peak width, charge distribution, and base peak intensity of model peptides (Figure S1); abundance of precursor charge states in peptide mapping of monoclonal antibody (Figure S2); peptide hydrophobicity- and pI-dependent change of AUC (Figure S3); effects of adding DMSO to PrA-containing mobile phase on total ion current and charge distribution in microflow analyses (Figure S4); peptide hydrophobicity-dependent change of retention time (Figure S5); dependence of retention behavior on peptide pI (Figure S6); dependence of peak broadening on peptide hydrophobicity in separation using HALO column (Figure S7); effects of alternative additives on peptide modification rate in the analysis of complex sample (Figure S8); relative concentrations of elements in treated mobile phase samples (Figure S9); effect of alternative additives on MS background noise (Figure S10) (PDF)

## Abbreviations

AcA: acetic acid
ACN: acetonitrile
AUC: area under curve
BRC: Biomedical Research Center
DMSO: dimethyl sulfoxide
ESI: electrospray ionization
FA: formic acid
FEP: fluorinated ethylene propylene
FPh: Faculty of Pharmacy in Hradec Králové
i.d.: internal diameter
IMB: Institute of Microbiology of the Czech Academy of Sciences in Prague
OPTIn: Organ Pathobiology and Therapeutics Institute, University of Pittsburgh
PrA: propionic acid
TFA: trifluoroacetic acid
