# Supplementary material for: Propionic Acid Outperforms Formic and Acetic Acid in MS Sensitivity for High-Flow Reversed-Phase LC-MS Bottom-Up Proteomics
Source: Anal Chem. 2026 Apr 3;98(14):10572–83. doi: 10.1021/acs.analchem.5c07595 (PMC13084630; doi:10.1021/acs.analchem.5c07595)
Supplement: Supplementary file 1 [file ac5c07595_si_001.pdf]

Supporting Information

Propionic acid outperforms formic and acetic acid in MS sensitivity for high-flow reversed-phase LC-MS bottom-up proteomics

Mykyta R. Starovoit[1‡], Siddharth Jadeja[1‡], Rudolf Kupčík[2], Saša Vatić[3], Jan Rasl[3,4], Derya Demir[1], Petr Novák[3,4], Cameron Braswell[5], Benjamin C. Orsburn[5], Juraj Lenčo[1*]

[1] Department of Analytical Chemistry, Faculty of Pharmacy in Hradec Králové, Charles University, Heyrovského 1203/8, 500 03 Hradec Králové, Czech Republic

[2] Biomedical Research Centre, University Hospital Hradec Králové, Sokolská 581, 500 05 Hradec Králové, Czech Republic

[3] Laboratory of Structural Biology and Cell Signaling, Institute of Microbiology, Czech Academy of Sciences, BioCeV, Vídeňská 1083, 142 00 Prague 4, Czech Republic

[4] Department of Biochemistry, Faculty of Science, Charles University, Hlavova 6, 12843 Prague 2, Czech Republic

[5] Organ Pathobiology and Therapeutics Institute, University of Pittsburgh, Pittsburgh, PA 15203, USA

[*]Corresponding Author: E-mail: lenco@faf.cuni.cz, Phone: +420 495 067 381.

[‡] These authors contributed equally to this work.

Table of Contents

Note S1: Sample preparation S3

Note S2: Search parameters for bottom-up LC-MS data S4

Note S3: GC-MS profiling S5

Table S1: Ion source settings S6

Table S2: Settings of MS1 and DDA/DIA experiments S7

Table S3: Concentration (ppb) of elements influenced by the selection of acidifier S8

Figure S1: Effects of alternative additives on peak width, charge distribution, and base peak intensity of model peptides S9

Figure S2: Abundance of precursor charge states in peptide mapping of monoclonal antibody S10

Figure S3: Peptide hydrophobicity- and pI-dependent change of AUC S11

Figure S4: Effects of adding DMSO to PrA-containing mobile phase on total ion current and charge distribution in microflow analyses S12

Figure S5: Peptide hydrophobicity-dependent change of retention time S13

Figure S6: Dependence of retention behavior on peptide pI S14

Figure S7: Dependence of peak broadening on peptide hydrophobicity in separation using HALO column S15

Figure S8: Effects of alternative additives on peptide modification rate in analysis of complex sample S16

Figure S9: Relative concentrations of elements in treated mobile phase samples S17

Figure S10: Effect of alternative additives on MS background noise S18

Supporting References S19

Note S1: Sample preparation

iRT and Alberta peptides (FPh)

Stock solutions of seven selected iRT peptides (LGGNEQVTR, GAGSSEPVTGLDAK, YILAGVENSK, TPVITGAPYEYR, ADVTPADFSEWSK, GTFIIDPGGVIR, and LFLQFGAQGSPFLK) and four Alberta peptides (acetyl-GGGLGGAGGLKG, acetyl-KYGLGGAGGLKG, acetyl-GGAVKALKGLKG, and acetyl-KYALKALKGLKG) were mixed so that the concentration of each was around 0.0125 µg/µL. The sample contained 20% acetonitrile, 0.1% TFA, and 0.001% PEG 20 000.

Trypsin digestion of a monoclonal antibody bevacizumab (FPh)

Bevacizumab was denatured in 5 M guanidinium chloride and reduced in 20 mM dithiothreitol for 30 min at room temperature. Thiols were not blocked. The mixture was diluted with Low-Artifact Digestion Buffer (Merck/Sigma-Aldrich) enriched with dithiothreitol, resulting in a concentration of 2 mM. The protein was digested using dimethylated SOLu-Trypsin at a 1:20 enzyme-to-protein ratio at 37 °C for 2 hours. The digest was acidified with TFA, filtered, and desalted using the Pierce Peptide Desalting spin column (Thermo Fisher Scientific). The desalted peptides were vacuum-dried and reconstituted in 0.1% TFA and 0.001% PEG 20 000.

Trypsin digestion of Jurkat cell proteins (FPh)

The Jurkat cells (ATCC TIB-152) were cultured in 150 cm^2 cultivation flasks (TPP) in RPMI 1640 medium supplemented with 10% fetal bovine serum. Cells were washed with phosphate-buffered saline and lysed on ice with 2.5% sodium deoxycholate containing 125 UI/mL benzonase. The protein concentration was determined using a bicinchoninic acid assay (Merck/Sigma Aldrich). One milligram of protein was buffered with 1 M Tris-HCl, pH 7.5, and reduced in 20 mM dithiothreitol for 30 min at room temperature. The free thiol groups were blocked using 50 mM chloroacetamide. The proteins were digested using dimethylated SOLu-trypsin at a 1:50 ratio overnight at 37 °C. The digest was acidified with TFA, and the precipitated deoxycholic acid was extracted using liquid-liquid extraction into ethyl acetate saturated with water. The supernatant was desalted using the Pierce Peptide Desalting spin columns. The desalted peptides were vacuum-dried and reconstituted in 0.1% TFA.

Reconstitution and desalting of digested proteins of HeLa cells (IMB)

The lyophilized Pierce™ HeLa Protein Digest Standard was reconstituted in 2% ACN and 0.1% TFA to a final concentration of 0.5 µg/µL. The reconstituted digest was subsequently diluted to working concentrations of 2, 10, 20, and 100 pg/µL in a buffer containing 0.1% FA and 0.1% n-dodecyl-β-D-maltoside, ensuring equal sample volumes for loading onto C_{18} solid-phase extraction tips (EvoTips, EvoSep Biosystems). The digest was loaded onto EvoTips according to the manufacturer's protocol, with the relative centrifugal force (RCF) adjusted and spin times extended to ensure complete passage of liquid through the tips. The washing and equilibration steps were performed as follows: ACN supplemented with 0.1% FA at 100 RCF, isopropanol at 150 RCF, and 0.1% FA at 150 RCF. Samples were loaded at 350 RCF and desalted with 100 µL of 0.1% FA at 150 RCF.

Note S2: Search parameters for bottom-up LC-MS data

Effects of propionic acid on peptide mapping of monoclonal antibody

The data were searched in Byonic v3.5.0 against the FASTA of bevacizumab downloaded from the Drug Bank and appended with common protein contaminants.[1, 2] The mass tolerance was 8 ppm for precursors and 18 ppm for fragments. A semitryptic cleavage with two missed cleavages was used. Oxidized Met, pyroGlu formation at N-terminal Glu and Gln, deamidated Asn, and dehydrated Asp were set as dynamic modifications. The search included a library of 132 human plasma N-glycans. Peptide-spectrum matches identified with a 2D FDR ≤ 1% were considered. For quantitative evaluation, MS1 peaks were extracted using Skyline.

Effects of propionic acid on analytical- and microflow analyses of complex samples

The data were searched in Proteome Discoverer v2.3 using Byonic v3.5.0. The spectra were searched against the human protein database downloaded from UniProt (UP000005640). The mass tolerance was 7.5 ppm for precursors and 20 ppm for fragments. A semitryptic cleavage with one missed cleavage was used. Acetylated protein N-terminus, Oxidized Met, pyroGlu formation at N-terminal Glu and Gln, deamidated Asn, and dehydrated Asp were set as dynamic modifications. Carbamidomethylation of Cys was set as a fixed modification. Peptide-spectrum matches identified with a 2D FDR ≤ 1% were considered. Additionally, the spectra were searched against the same protein database using MSFragger, integrated into Skyline via the Peptide Search workflow, with recalibration of spectra to access peptide-specific spectral information. The cutoff score was set at 0.95. A fully specific cleavage with no missed cleavages was used. Possible precursor charges 2, 3, 4, and 5 were considered. The mass tolerance was 5 ppm for precursors and 15 ppm for fragments. Chromatograms from three monoisotopic peaks were extracted within 10 min around the identification time.

Effects of propionic acid on nanoflow analyses of complex samples

At BRC, the data were treated as described in the previous section.

At OPTIn, the data were subjected to automatic processing with a command-line script in HyStar using DIA-NN 1.9.1. Briefly, a predicted library containing 21,200 human proteins (UniProt-reviewed, downloaded April 2025) was appended with the cRAP contaminant database (www.gpm.org) for comparison. Static carbamidomethylation of cysteines was considered, and methionine oxidation was included as a dynamic modification. Terminal protein methionine cleavage was also considered a dynamic modification, as well as the possibility of up to two missed cleavage events. Each file was analyzed separately with this workflow, with no files combined through tools such as match between runs. The DIA-NN summary output sheets were then used for all subsequent statistical analyses.

At IMB, raw data were processed automatically using a script provided by Bruker Daltonics within the HyStar environment and analyzed with DIA-NN (version 1.9.2). Both the Swiss-Prot human FASTA database (containing 42,531 protein entries, downloaded in October 2021) and the spectral library used for spectral matching were supplied by Bruker Daltonics. The database search included a fixed modification of cysteine residues (carbamidomethylation) and variable modifications of methionine oxidation, asparagine/glutamine deamidation, and lysine acetylation. Each sample was analyzed independently. The resulting DIA-NN Parquet files were used for downstream data analysis.

Note S3: GC-MS profiling

The column was a 30 m × 0.25 mm Agilent HP-5MS with 0.25 µm film thickness. The carrier gas was helium at a constant flow rate of 0.7 mL/min. The injector inlet was in splitless mode, and the heater temperature was set to 260 °C. The injection volume was 1 µL. The column temperature was maintained at 40 °C for 1 min, then increased to 100 °C at a rate of 15 °C/min, followed by a further increase to 210 °C at a rate of 10 °C/min. Finally, the temperature was increased to 300 °C at a rate of 5 °C/min and held for 8 min. After each analysis, the temperature returned to 40 °C and was held to equilibrate for additional 3 min. The temperature of the AUX transfer line was set at 300 °C. The mass spectra were obtained in the m/z range of 30-1050.

Table S1: Ion source settings

Parameter	Q Exactive HF-X, analytical-/microLC, **FPh**					
	Model peptides	Digest of monoclonal antibody	Digest of Jurkat cell proteins			Direct infusion MS
Column internal diameter, mm	2.1	2.1	1.0	1.5	2.1	no column
Mobile phase flow rate, µL/min	300	250	50	125	250	25
**Ion Source Parameter**						
Sheath gas flow rate	35.0	25.0	30.0	38.0	48.0	21.0
Auxiliary gas flow rate	10.0	10.0	10.0	10.0	11.0	7.0
Sweep gas flow rate	2.0	2.0	1.0	1.0	2.0	0.0
Spray voltage, kV	3.0	3.5	3.5	3.5	3.5	2.5
Capillary temperature, °C	275.0	275.0	250.0	250.0	256.0	250.0
Aux gas heater temperature, °C	450.0	325.0	151.0	250.0	413.0	50.0
Depth of the ESI probe	halfway between B-C		A	B	halfway between B-C	A

Parameter	Q Exactive Plus microLC, **BRC**	Orbitrap Exploris 480 nanoLC, **BRC**	timsTOF SCP, nanoLC, **IMB**
Column internal diameter, mm	1.0	0.075	0.075
Mobile phase flow rate, µL/min	68	0.25	0.2
**Ion Source Parameter**			
Sheath gas flow rate	25	X	3 L/min
Auxiliary gas flow rate	6	X	X
Sweep gas flow rate	1	X	X
Spray voltage, kV	3000	1500	1300
Capillary temperature, °C	275	X	200
Aux gas heater temperature, °C	200	X	X
Depth of the ESI probe	C	X	X

Table S2: Settings of MS1 and DDA/DIA experiments

	Model peptides, **FPh**	Direct infusion MS, **FPh**
MS1 settings		
Polarity	Positive	Positive/negative
Resolution at 200 m/z	45 000	240 000
Automatic gain control target	3×10^6	3×10^6
Maximum injection time	86 ms	100 ms
Scan range	150 to 1500 m/z	50 to 750 and 400 to 6000 m/z

**DDA experiments**	Digest of monoclonal antibody, **FPh**	Digest of Jurkat cell proteins, **FPh**	Digest of HeLa cell proteins, microLC, **BRC**	Digest of HeLa cell proteins, nanoLC, **BRC**
MS1 settings				
Resolution at 200 m/z	60 000	60 000	35 000	120 000
Automatic gain control target	1×10^6	3×10^6	3×10^6	300 % (normalized)
Maximum injection time	118 ms	110 ms	110 ms	Auto
Scan range	300 to 1500 m/z	350 to 1500 m/z	350 to 1600 m/z	350 to 1500 m/z
DDA and MS2 settings				
Max. number of precursors	3	10	12	10
Precursor charge states	2, 3, 4, 5	2, 3, 4, 5	2, 3, 4, 5, 6, 7	2, 3, 4, 5
Intensity threshold	2.5×10^5	1×10^5	8.3×10^3	1×10^5
Isolation window	2.5 m/z	1.8 m/z	2.0 m/z	2.0 m/z
Isolation offset	0.3 m/z	0.3 m/z	Off	Off
Normalized collision energy	27	27	28	28
Resolution at 200 m/z	30 000	15 000	17 500	30 000
Automatic gain control target	2×10^5	2×10^5	1×10^6	100% (normalized)
Maximum injection time	100 ms	50 ms	120 ms	200 ms
Isotopes exclusion	On	On	On	On
Dynamic exclusion time	3.0 s	30.0 s	30.0 s	20.0 s
Apex trigger	1 to 3 s	Off	Off	Off

**DIA experiments**	
Digest of K-562 cell proteins<br>nanoLC, **OPTIn**	LC-MS DIA data were acquired with the vendor's default short gradient high-sensitivity diaPASEF method. Ion mobility separation was set with $1/k_0$ limits of 0.64–1.50. Each acquisition cycle was completed in 0.96 s, consisting of one MS1 survey ramp followed by 8 MS/MS ramps. Fragmentation was carried out across 24 diaPASEF windows, covering a precursor mass range of 400–1000 m/z in 25 Da increments, with corresponding $1/k_0$ values of 0.64-1.37.
Digest of HeLa cell proteins<br>nanoLC, **IMB**	DIA data were acquired in positive ion mode using a diaPASEF method over an m/z range of 100-1700 and an ion mobility range of 0.7-1.6 V·s/cm². Each acquisition cycle consisted of one MS1 ramp followed by 16 MS2 ramps covering 32 PASEF windows spanning 400–1200 m/z and 0.8-1.3 $1/k_0$. Each MS2 window had a width of 25 m/z, and the total cycle time was 2.94 s.

Table S3: Concentration (ppb) of elements influenced by the selection of acidifier

	FA ctrl		PrA ctrl		FA glass		PrA glass		FA circ		PrA circ	
**Element**	Mean	SD	Mean	SD	Mean	SD	Mean	SD	Mean	SD	Mean	SD
**Na**	<10		<10		<10		<10		<10		<10	
**K**	<55		<55		<55		<55		<55		<55	
**Ca**	<150		<150		<150		<150		<150		<150	
**Fe**	< 0.40		< 0.40		< 0.40		< 0.40		50,18	1,31	158,95	3,38
**B**	38,23	3,70	29,90	0,87	33,61	1,17	35,91	3,23	52,30	4,65	52,16	6,10
**Cr**	< 0.25		< 0.25		< 0.25		< 0.25		14,53	0,10	35,04	0,36
**Zn**	16,02	0,48	18,74	2,29	24,30	0,62	20,34	1,06	23,33	1,33	33,28	2,94
**Mg**	26,60	0,59	33,29	4,94	29,00	1,00	30,19	2,47	23,47	1,61	25,66	3,41
**Ni**	0,40	0,04	0,48	0,08	0,45	0,09	2,09	0,23	4,21	0,19	13,73	0,26
**Cu**	0,49	0,01	0,27	0,03	2,85	0,10	2,12	0,10	2,53	0,18	8,58	0,29
**Al**	5,20	0,56	13,19	1,39	13,39	1,14	6,42	0,92	15,91	3,16	7,29	0,82
**Mo**	0,08	0,00	0,05	0,00	0,06	0,00	0,07	0,00	1,00	0,01	3,02	0,09
**Pb**	0,16	0,01	0,07	0,01	1,20	0,06	0,15	0,00	0,88	0,02	2,96	0,03
**Mn**	0,24	0,01	0,22	0,02	0,22	0,03	0,26	0,02	1,22	0,01	2,21	0,15
**Cd**	0,02	0,00	0,03	0,00	0,02	0,00	0,01	0,00	2,47	0,03	1,84	0,04
**Ba**	0,08	0,01	0,39	0,08	0,19	0,01	0,19	0,01	1,02	0,00	0,89	0,02
**Ti**	0,11	0,01	0,11	0,00	0,10	0,01	0,10	0,01	0,30	0,02	0,34	0,03
**Co**	0,00	0,00	0,00	0,00	1,24	0,05	10,15	0,05	0,23	0,00	0,32	0,02

	ACN Fischer Chem.	Water VWR	ACN Honeywell	ACN Supelco	Water Supelco	Waters Guideline[3]
**Element**	Maximum allowed concentration					
**Na**	10	50	50	50	200	50
**K**	10	50	50	5	10	50
**Ca**	10	50	50	10	100	50
**Fe**	5	50	20	10	5	10-20
**B**	N/A	N/A	N/A	N/A	N/A	N/A
**Cr**	2	N/A	20	5	5	N/A
**Zn**	5	N/A	100	5	5	N/A
**Mg**	5	50	100	10	20	10-20
**Ni**	2	N/A	N/A	5	5	N/A
**Cu**	5	N/A	N/A	5	5	N/A
**Al**	5	50	500	10	10	25-50
**Mo**	N/A	N/A	100	5	5	N/A
**Pb**	5	N/A	20	5	5	10-20
**Mn**	2	N/A	20	5	5	N/A
**Cd**	2	N/A	50	5	5	N/A
**Ba**	2	N/A	100	5	5	N/A
**Ti**	N/A	N/A	N/A	5	5	N/A
**Co**	2	N/A	20	5	5	N/A

The second part of the table lists the maximum allowed concentrations by selected manufacturers in their LC-MS solvents.

Figure S1: Effects of alternative additives on peak width, charge distribution, and base peak intensity of model peptides

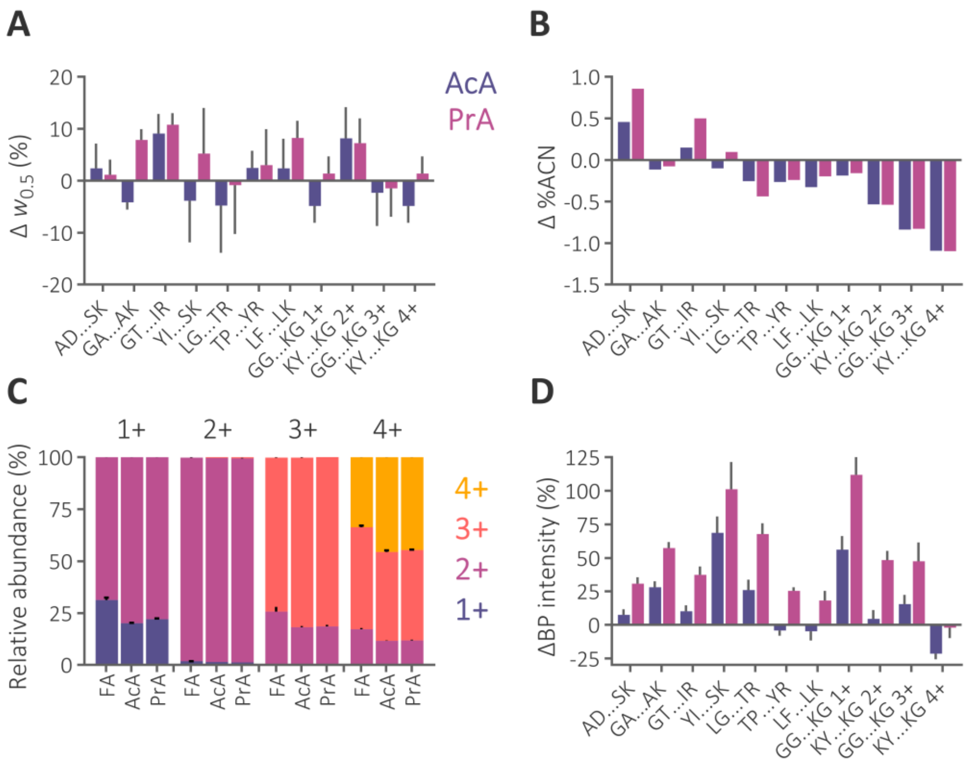

E

Set	Sequence	Monoisotopic mass	pI	Net charge at pH 2.7	t_R
	ADVTPADFSEWSK	1451,657	3,7	0,9	7,54
	GAGSSEPVTGLDAK	1287,631	4,1	1,0	4,83
	GTFIIDPGGVIR	1243,692	6,9	1,0	8,72
**iRT**	YILAGVENSK	1092,582	6,9	1,0	5,78
	LGGNEQVTR	972,499	6,9	1,0	3,27
	TPVITGAPYEYR	1365,693	7,0	1,0	6,44
	LFLQFGAQGSPFLK	1551,845	10,1	1,1	9,83
	acetyl-GGGLGGAGGLKG	941,493	6,4	0,5	6,04
**Alberta**	acetyl-KYGLGGAGGLKG	1118,608	10,1	1,2	5,56
	acetyl-GGAVKALKGLKG	1139,703	10,9	2,2	5,37
	acetyl-KYALKALKGLKG	1330,834	10,7	3,2	5,69

(A) Relative change of chromatographic peak width at half height ($w_{0.5}$) of peptides from iRT and Alberta sets separated using a 2.1 × 150 mm Acquity Premier CSH C_{18} column and mobile phases containing 0.5% AcA or 0.5% PrA in comparison to the separation using 0.1% FA. iRT peptides are listed from left to right in order of increasing isoelectric points. The number of chargeable amino groups is indicated for Alberta peptides. **(B)** Relative change of retention time of peptides from iRT and Alberta sets expressed as the apparent ACN content at elution, calculated from the linear gradient by converting retention time to the corresponding mobile phase composition. **(C)** Relative abundance of Alberta peptides precursors generated in the ion source after separation using mobile phases containing 0.1% FA, 0.5% AcA, and 0.5% PrA. The number of lysine residues in peptide sequences is indicated above. The colored legend corresponds to precursor charge states. **(D)** Relative change of base peak (BP) intensity of iRT and Alberta peptides. **(E)** Properties of iRT and Alberta peptides. Isoelectric points and net charges at pH 2.7 corresponding to aqueous 0.1% FA were calculated using Bachem Peptide Calculator (Bachem AG, Switzerland). Retention times were extracted from the analyses using the mobile phase containing 0.1% FA.

Figure S2: Abundance of precursor charge states in peptide mapping of monoclonal antibody

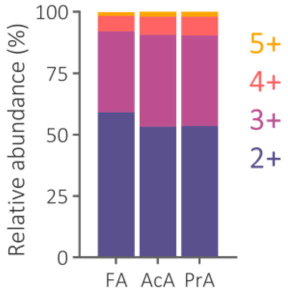

Average relative abundance of precursor charge states generated in the ion source after separation of bevacizumab peptides using mobile phases containing 0.1% FA, 0.5% AcA, and 0.5% PrA.

Figure S3: Peptide hydrophobicity- and pI-dependent change of AUC

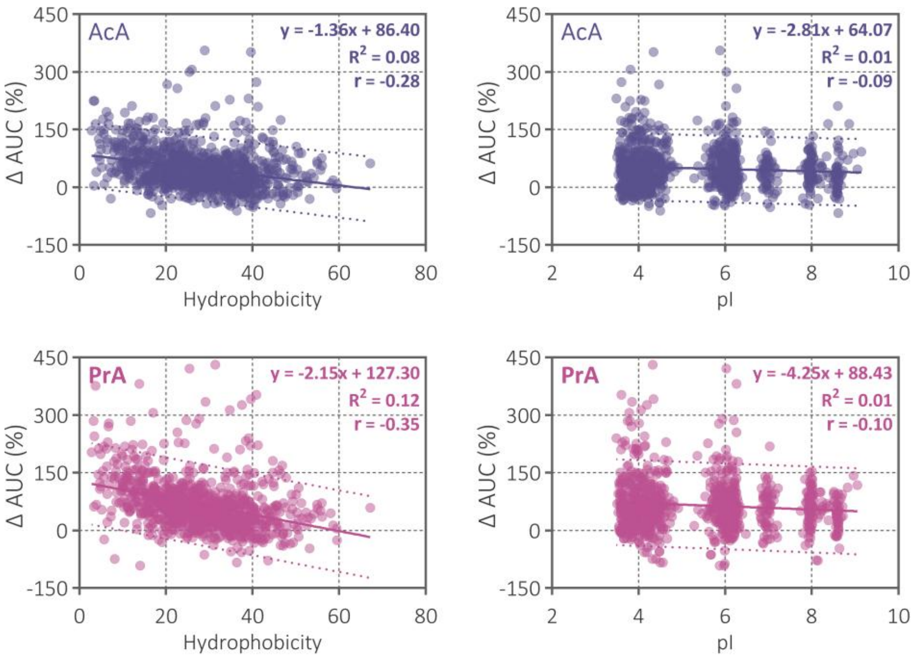

Relative AUC change of 955 peptides identified in separations using AcA- and PrA-containing mobile phases in comparison to FA on the peptide hydrophobicity and isoelectric point. The peptide set contains unique unmodified peptides of 6-25 amino acids in length, with RSD of AUC in each additive dataset under 50%, compiled from pooled identifications across six column datasets.

Figure S4: Effects of adding DMSO to PrA-containing mobile phase on total ion current and charge distribution in microflow analyses

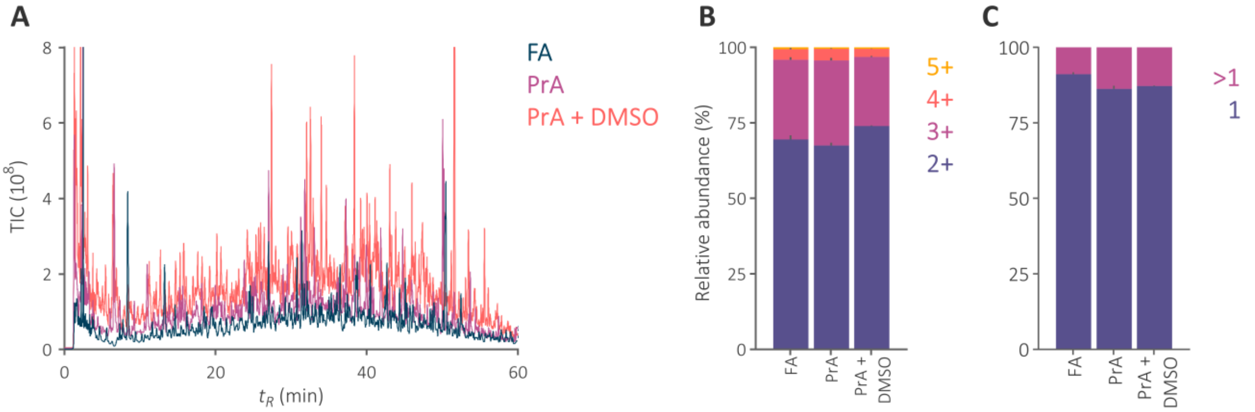

(A) Total ion current (TIC) chromatograms of analyses of digested Jurkat cell proteins using mobile phases containing 0.1% FA, 0.5% PrA, and 0.5% PrA with 3% DMSO. **(B)** Relative abundance of precursor charges in peptide-spectrum matches. **(C)** Relative abundance of peptides identified with a single (1) and multiple precursors (>1).

Figure S5: Peptide hydrophobicity-dependent change of retention time

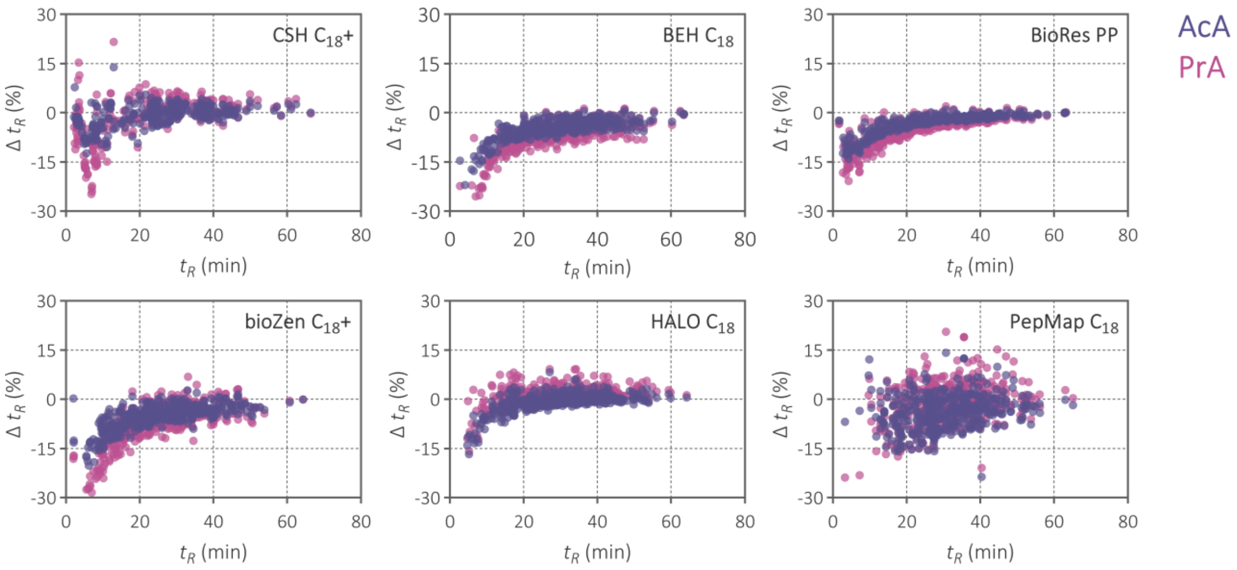

Dependence of relative retention time change of 400 peptides separated on different columns using AcA- and PrA-containing mobile phases in comparison to FA on the retention time observed in the analyses using FA.

Figure S6: Dependence of retention behavior on peptide pI

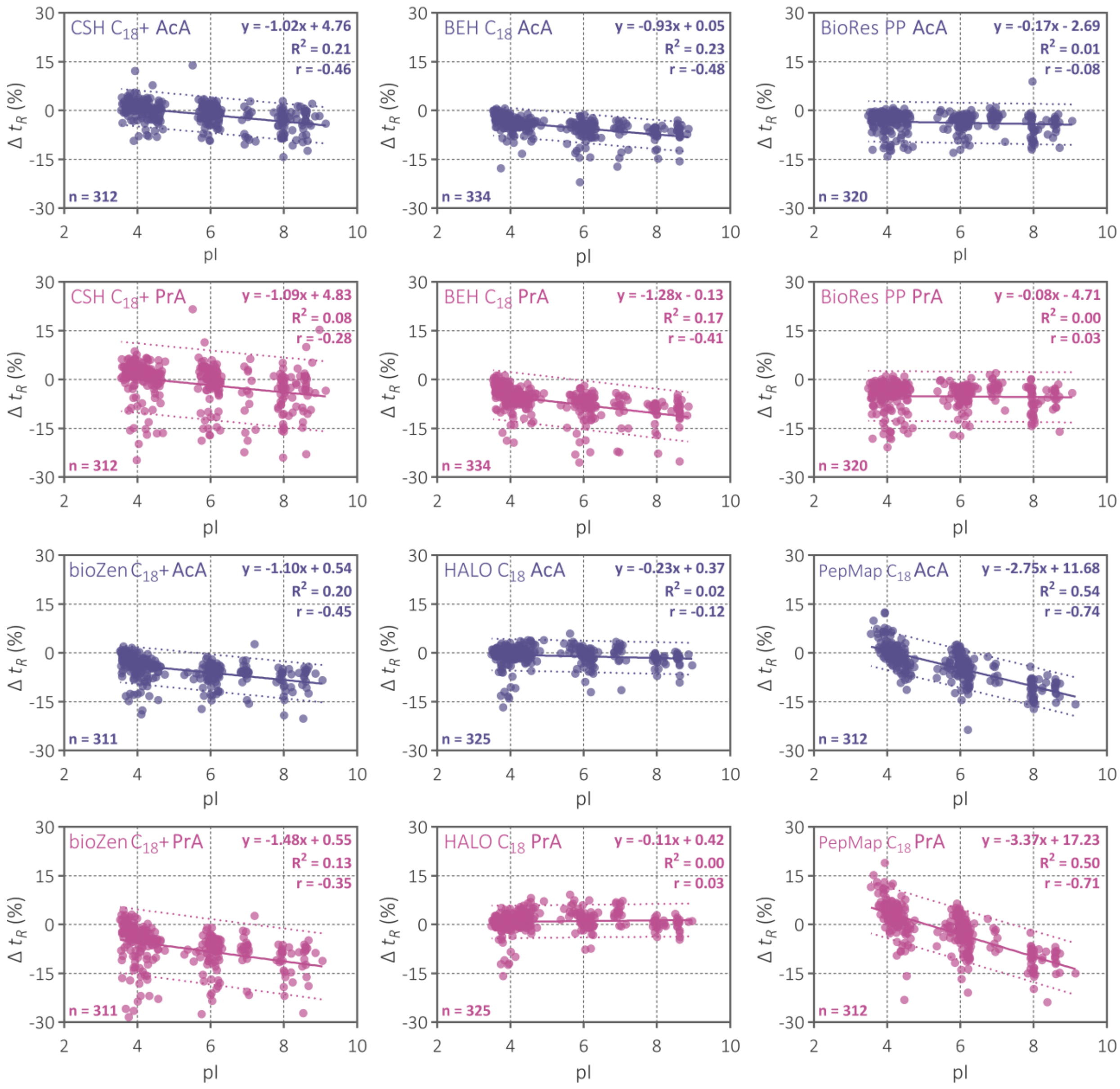

Dependence of relative retention time change when separated on different columns using AcA- and PrA-containing mobile phases in comparison to FA on the peptide isoelectric points with linear regression. Columns, mobile phase additives, linear regression equations, determination coefficients, Pearson correlation coefficients, and the number of selected unmodified peptides are shown. Colored dots illustrate 90% prediction bands.

Figure S7: Dependence of peak broadening on peptide hydrophobicity in separation using HALO column

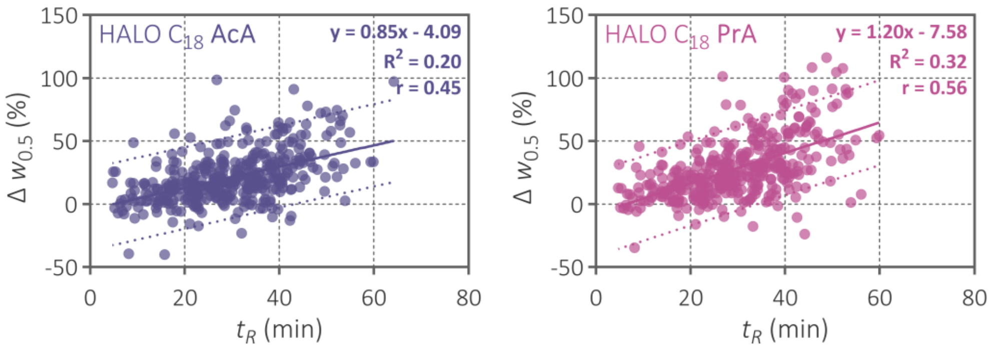

Dependence of peak width change on retention time of 400 peptides separated on the 1.5 × 150 mm HALO 160 Å ES-C_{18} column using AcA- and PrA-containing mobile phases in comparison to FA in analyses using 0.1% FA with linear regressions and Pearson correlation coefficients.

Figure S8: Effects of alternative additives on peptide modification rate in analysis of complex sample

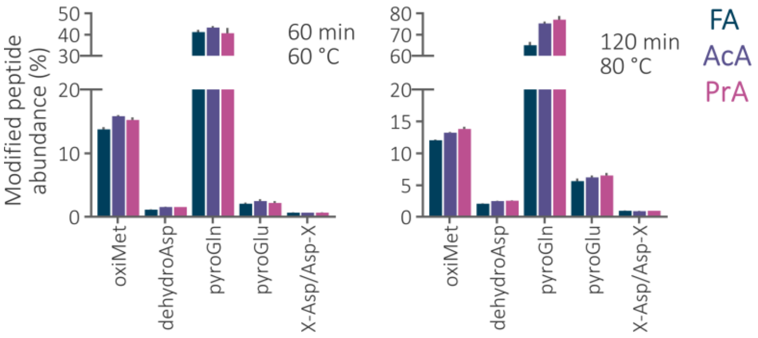

Relative abundance of modified peptides among all the identified peptides containing the modified amino acid in the 20 and 90 min separations of Jurkat cell peptides using the 1.0 × 150 mm Acquity UPLC CSH C18 column maintained at 60 and 80 °C, respectively, and FA-, AcA-, and PrA-containing mobile phases.

Figure S9: Relative concentrations of elements in treated mobile phase samples

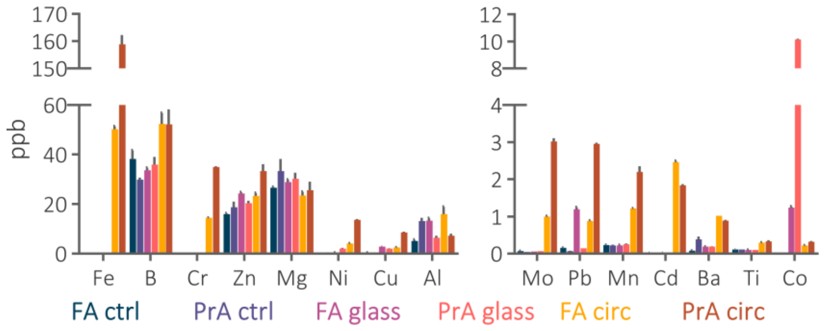

Relative concentrations of elements contained in the mobile phases after incubation in inert (*ctrl*) and glass (*glass*) containers or circulation in the LC system (*circ*). Only the elements with significant changes are plotted.

Figure S10: Effect of alternative additives on MS background noise

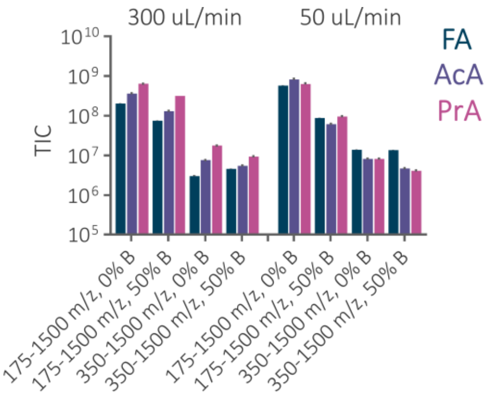

Total ion currents observed under 0.1% FA, 0.5% AcA, and 0.5% PrA conditions at the flow rates of 50 and 300 µL/min in the m/z ranges of 175-1500 and 350-1500, infusing either 0% or 50% component B of the mobile phase. The *y*-axis uses a decadic logarithmic scale.

Supporting References

(1) Wishart, D. S.; Feunang, Y. D.; Guo, A. C.; Lo, E. J.; Marcu, A.; Grant, J. R.; Sajed, T.; Johnson, D.; Li, C.; Sayeeda, Z.; et al. *Nucleic Acids Res* **2018**, *46*, D1074-D1082.
(2) Bern, M.; Kil, Y. J.; Becker, C. *Curr Protoc Bioinformatics* **2012**, *Chapter 13*, 13 20 11-13 20 14.
(3) Waters. *User Manual* **2023**, *EN715009076*.
